# Beamline B21: high-throughput small-angle X-ray scattering at Diamond Light Source

**DOI:** 10.1107/S1600577520009960

**Published:** 2020-08-18

**Authors:** Nathan P. Cowieson, Charlotte J. C. Edwards-Gayle, Katsuaki Inoue, Nikul S. Khunti, James Doutch, Eugene Williams, Steven Daniels, Geoff Preece, Nicholas A. Krumpa, John P. Sutter, Mark D. Tully, Nick J. Terrill, Robert P. Rambo

**Affiliations:** a Diamond Light Source Ltd, Harwell Science and Innovation Campus, Didcot, Oxfordshire OX11 0DE, United Kingdom; b ISIS Neutron and Muon Source, Science and Technology Facilities Council, Rutherford Appleton Laboratory, Didcot, Oxfordshire OX11 0QX, United Kingdom; c Projects and Mechanical Engineering Group, Science and Technology Facilities Council, Daresbury Laboratory, Warrington, Cheshire WA4 4AD, United Kingdom; d BM29 BIOSAXS, European Synchroton Radiation Facility, 71 avenue des Martyrs, Grenoble, Isère 38043, France

**Keywords:** SAXS, proteins, biological macromolecules, solution samples

## Abstract

B21 is a small-angle X-ray scattering (SAXS) beamline with a bending magnet source in the 3 GeV storage ring at the Diamond Light Source Ltd synchrotron in the UK. The beamline utilizes a double multi-layer monochromator and a toroidal focusing optic to deliver 2 × 10^12^ photons per second to a 34 × 40 µm (FWHM) focal spot at the in-vacuum Eiger 4M (Dectris) detector. A high-performance liquid chromatography system and a liquid-handling robot make it possible to load solution samples into a temperature-controlled in-vacuum sample cell with a high level of automation. Alternatively, a range of viscous or solid materials may be loaded manually using a range of custom sample cells. A default scattering vector range from 0.0026 to 0.34 Å^−1^ and low instrument background make B21 convenient for measuring a wide range of biological macromolecules. The beamline has run a full user programme since 2013.

## Abstract 2

The B21 beamline at Diamond Light Source is optimized for automated measurement of small-angle X-ray scattering from solution samples.

## 1. Introduction   

The bending magnet small-angle X-ray scattering beamline B21 at Diamond Light Source is designed to facilitate measurement of scattering data from solution samples. The beamline is most commonly in a default configuration with a beam energy of 13 keV, a sample-to-detector distance of 3.7 m and an in-vacuum sample cell to which solution samples are delivered by either a liquid-handling platform or an in-line high-performance liquid chromatography (HPLC) system. This setup gives a scattering vector (*Q*) range from 0.0026 to 0.34 Å^−1^ (*d* spacing from 2400 to 19 Å), useful for characterizing a wide range of samples including many commonly studied biological macromolecules and complexes.

B21 operates with a high-throughput approach to SAXS experiments that allows rapid turnover between different user groups. This is achieved using a largely static instrument configuration, *i.e.* fixed camera length, sample environment and X-ray energy, that minimizes significant reconfigurations between experiments. In addition, having a single sample environment facilitates a high level of integration and automation. Other SAXS beamlines, such as BM29 at the ESRF (Pernot *et al.*, 2010 ▸), P12 at DESY (Blanchet *et al.*, 2015 ▸) and SIBYLS at the ALS (Classen *et al.*, 2013 ▸), have a similar design ethos with greater or lesser options for reconfiguration between experiments.

By contrast, the more general purpose SAXS beamlines such as I22 at Diamond Light Source (Smith *et al.*, 2019 ▸), SWING at SOLEIL (David & Pérez, 2009 ▸) and the SAXS/WAXS beamline at the Australian Synchrotron (Kirby *et al.*, 2013 ▸) are designed to cater for a wide range of sample environments and beamline configurations where user experiments require significant setup times, albeit at the cost of user and sample throughput. Such setup costs can be offset by engineering innovative modular sample environments that facilitate rapid changing and alignment.

B21 has run a user programme since 2013 that has steadily built up to 240 individual user group visits in 2019 using 3535 h of beam time. There have been a total of 188 peer reviewed publications since 2013, with 60 publications from the beamline in 2019. The B21 beamline will be described in overview. Some highlights from the user programme are included.

## 2. Beamline overview   

**Figure 1:**
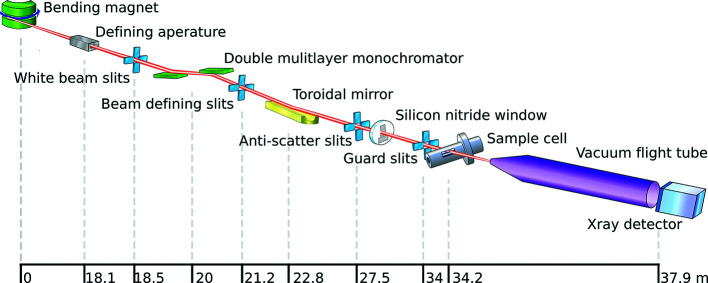
The layout of B21 with the source-to-element distances.

B21 is a Phase III Diamond Light Source bending magnet beamline with a multilayer monochromator (Axilon, Germany) and a toroidal focusing mirror (Winlight System, France). Four sets of slits are arranged to minimize instrumentation background scattering. Our single sample environment is provided by the Arinax BIOSAXS sample-handling robot which holds a modular in-vacuum sample cell that can be readily changed. Samples are loaded into the sample cell via an in-line HPLC, a liquid-handling robot or manually. A schematic layout of B21 can be seen in Fig. 1 ▸ and beamline details are given in Table 1 ▸.

### 2.1. Beamline optics and hardware   

#### 2.1.1. Source   

B21 has a 1.44 T bending magnet source in the 3 GeV storage ring of the Diamond Light Source synchrotron. The source size is 54.2 × 15.4 µm (horizontal × vertical) and it has a divergence of 122.7 × 1.5 µrad (RMS).

#### 2.1.2. Monochromator   

A double multilayer monochromator (DMM), positioned 20 m from the source, provides monochromatic X-rays within the range of 9 to 14.5 keV. The DMM was optimized to provide maximum flux within a physically constrained space using two flat Mo/B_4_C multilayer substrates (Rigaku) each with 250 × 2.4 nm layers, giving a bandpass of 0.8% and reflectivity of 81% and 62% at 14.5 and 9 keV, respectively. Note that while reflectivity generally falls with higher energy, in this case absorption leads to lower apparent reflectivity at 9 than at 14.5 keV.

#### 2.1.3. Focusing   

**Figure 2:**
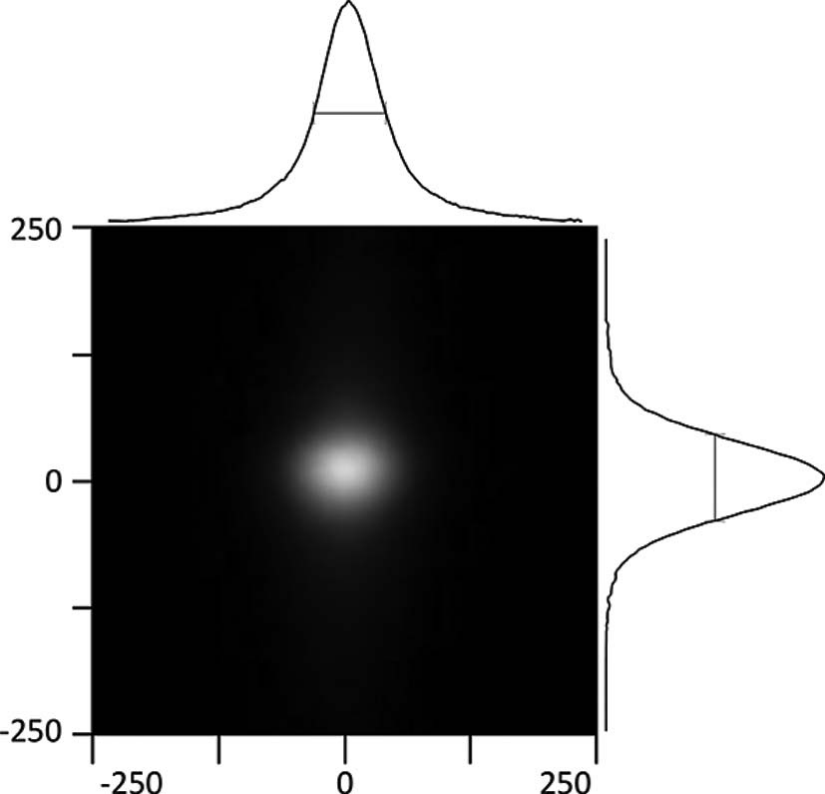
The focused beam at the detector position. The axes are labelled in micrometres.

Horizontal and vertical focus is achieved by a rhodium-coated silicon toroidal mirror at 23 m. The mirror has a reflecting surface 1100 mm long in the direction of the beam by 40 mm wide, a major radius of 6.455 km and a minor radius of 4.48 cm, giving a focal spot at the detector face (38.2 m) of around 50 µm (FWHM) (Fig. 2 ▸).

#### 2.1.4. Slit geometry   

**Figure 3:**
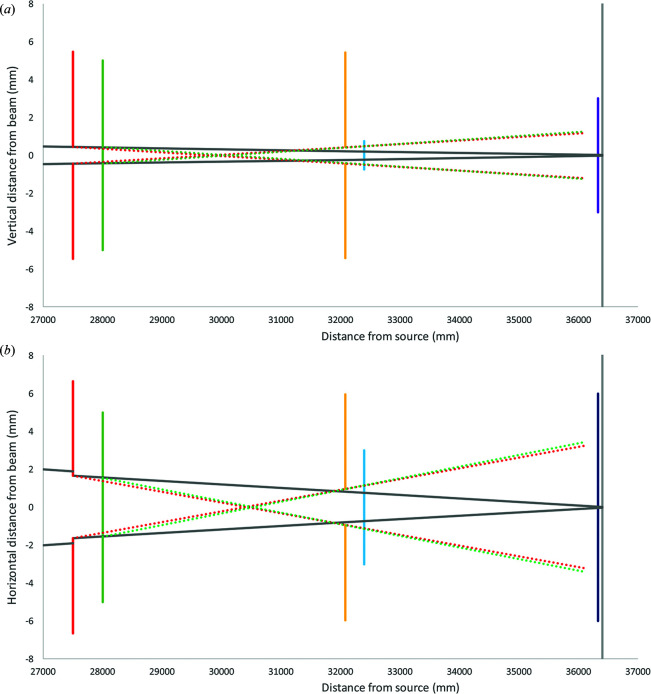
The slit geometry and scatter close to the sample, (*a*) in the vertical direction and (*b*) in the horizontal direction, showing the antiscatter slits (red), silicon nitride window (green), guard slits (yellow), sample (blue), beamstop (purple) and detector face (grey). The projections of scatter from the guard slit and silicon nitride window through the antiscatter slits are shown as red and green dotted lines, respectively.

The B21 X-ray beam is defined by four pairs of slits. The white-beam slits upstream of the DMM at 18.5 m (all distances mentioned are from the source) serve to fill the first multilayer with white beam from the source via the front end mask. A pair of slits upstream of the focusing optic at 21.2 m serve as the beam-defining slits. The vertical acceptance is limited by the portion of the cylindrical tangential bend of the focusing optic that produces well focused beam, and the horizontal acceptance is limited by the 10 × 10 mm silicon nitride window at 28 m. The beam-defining slits serve to fill this acceptance limit. A set of germanium-edged slits at 27.5 m act as antiscatter slits. A final set of germanium-edged antiscatter guard slits at 34 m are located upstream of the sample (∼200 mm) and serve to reduce the effect of scatter from the guard slits. Projections of the parasitic scatter from the upstream slits and silicon nitride window through the guard slits can be seen in Fig. 3 ▸. This simple geometry has been shown to be optimal for low-background X-ray scattering beamlines (Kirby *et al.*, 2013 ▸).

#### 2.1.5. X-ray camera   

**Figure 4:**
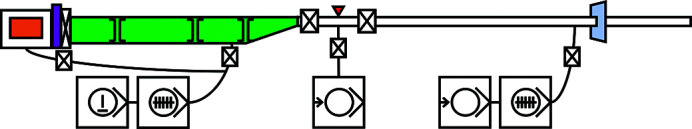
A schematic diagram of the endstation vacuum system, showing the detector (orange), beamstop module (purple), flight tubes (green) with moveable sections indicated by opposing square brackets, silicon nitride window (pale blue) and sample position (red triangle). Gate valves are indicated by rectangles with a cross through them. Pumps are indicated by squares with a circle and a right-facing arrow; roots pumps have two perpendicular lines inside the centre circle, turbomolecular pumps have a horizontal line with multiple cross hatches, and scroll pumps have a small arrow on the left-hand side.

The X-ray camera consists of a first-generation Eiger 4M (Dectris) detector that is fully enclosed inside a vacuum chamber. The detector vessel and an in-house designed beamstop assembly can be isolated from the camera vacuum tubes by means of a large gate valve, and the detector and beamstop module can be maintained under vacuum while being moved back and forth parallel to the beam to allow for changes to the sample-to-detector distance (Fig. 4 ▸). With the detector isolated and under vacuum, the flight tube part of the camera can be quickly vented to atmosphere and reconfigured using a combination of 2, 1 and 0.5 m sections of tubing. The distance between the detector and the sample position can thus be adjusted in increments of 0.5 m between 1.5 m (*Q* range 0.005 to 0.78 Å^−1^) and 4 m (*Q* range 0.002 to 0.3 Å^−1^). The focus of the beam at the detector face can be maintained by adjustment of the toroidal mirror’s pitch angle and tangential bending radius. Camera length changes can take a few hours and in practice B21 typically operates with a fixed camera length of 3.5 m and beam energy of 13.1 keV, giving a *Q* range from 0.0026 to 0.34 Å^−1^. Any changes to the camera length must be strongly supported by the experiment. Operating the detector in a vacuum removes the need for an exit window and allows the beamstop to be positioned around 3 mm from the surface of the detector. The close proximity of the beamstop to the detector contains the parasitic scatter and projection of the beamstop as close to the beam centre as possible.

The beamstop consists of a single silicon diode placed on a 10 × 3 mm stadium-shaped tungsten support with a concave front face to contain scatter. The beamstop is attached to an actuated beamstop assembly through a 2 mm diameter carbon fibre tube. The beamstop assembly provides limited translational motions, and yaw and pitch controls for fine tuning of the orientation of the beamstop.

### 2.2. Experimental parameters   

**Figure 5:**
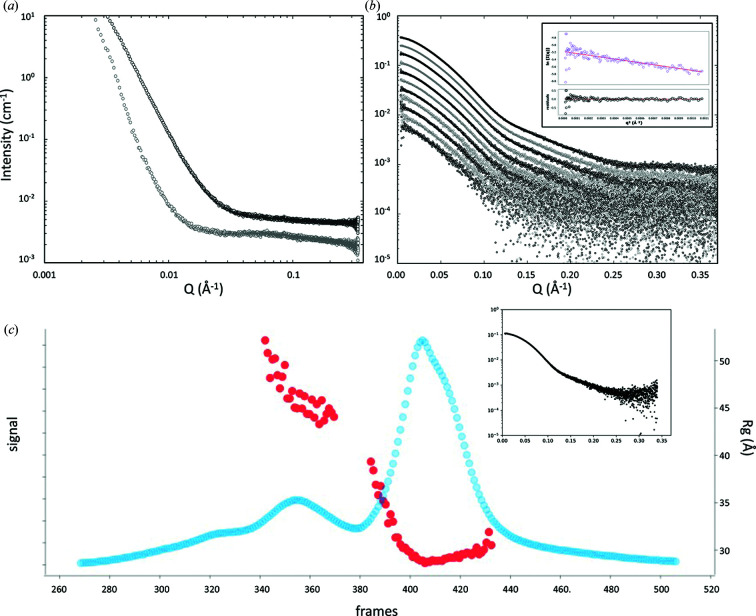
Example scattering data calibrated to absolute intensity relative to water (0.0163 Å^−1^). (*a*) Instrument background, measured with no capillary at the sample position (grey) and with an empty capillary (black). (*b*) An extended bovine serum albumin (BSA) dilution series measured at concentrations of 6, 4, 2.6, 1.7, 1.2, 0.8, 0.5, 0.35. 0.2, 0.13 and 0.08 mg ml^−1^ from top to bottom. The inset shows the Guinier fitting and residuals for the 0.08 mg ml^−1^ sample. For each dilution, 20 × 1 s frames were measured from 30 µl samples of BSA in 50 m*M* Tris pH 7.5, 150 m*M* NaCl, 2% glycerol at 15°C. (*c*) An SEC-SAXS trace of BSA. Blue points plot the magnitude of the scattering signal defined as the ratio of each frame to a background frame. The radius of gyration (red) is shown calculated for individual frames with scattering signal above a threshold. The inset shows scattering data derived by averaging frames across the main peak. The data represent a 50 µl sample of BSA at 5 mg ml^−1^ run on a KW403 column (Shodex) at 0.16 ml min^−1^ with a similar running buffer as described for panel (*b*).

The beam at the sample position is rectilinear (dimensions 1.1 mm horizontally and 0.24 mm vertically) and has a total flux of 2 × 10^12^ photons per second. The instrumentation scattering that contributes to background has been optimized to be as low as possible (Kirby *et al.*, 2013 ▸) [Fig. 5 ▸(*a*)], allowing protein samples to be measured with concentrations as low as 0.1 mg ml^−1^ [Fig. 5 ▸(*b*)].

### 2.3. Sample handling   

**Figure 6:**
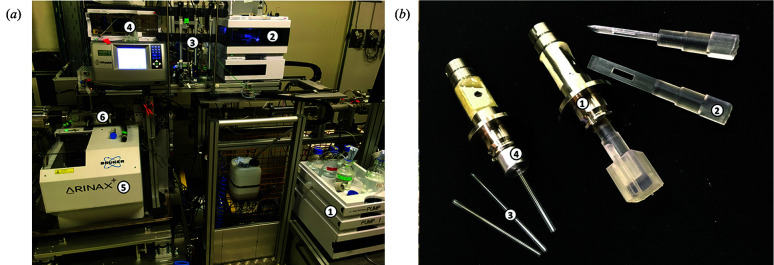
(*a*) The environment surrounding the sample cell, showing (1) an HPLC pump, (2) the HPLC autosampler, (3) a selection of columns, (4) the UV–VIS spectrometer, (5) the BIOSAXS robot and (6) the sample exposure unit. (*b*) The multi-purpose sample cell, showing (1) the cell body that fits in the BIOSAXS robot sample exposure unit, (2) a 3D-printed sample stick, (3) capillaries and (4) an adaptor for capillaries inside an MPS cell.

The B21 sample environment [Fig. 6 ▸(*a*)] supports three common ways of loading samples, namely automated-batch, HPLC and manual-loading mode.

#### 2.3.1. Liquid-handling robot   

The B21 sample environment is derived from the Arinax BIOSAXS liquid sample-handling robot (SHR) (Round *et al.*, 2015 ▸) that provides a sample viewing camera perpendicular to the X-ray beam and holds the temperature-controlled sample exposure unit (SEU) [Fig. 6 ▸(*a*), (6)]. The SEU holds a sample exposure pod which is a modular component that can be readily exchanged. Samples are loaded into the SEU from the sample storage unit (SSU) [Fig. 6 ▸(*a*), (5)], a temperature-controlled (4 to 40°C) environment where samples are arrayed in either 96-well plates, PCR strips or 1.5 ml microfuge tubes. Samples can then be transferred from the SSU to the SEU, which has two types of exposure mode, static mode and flow mode. Flow mode moves the sample through the capillary at a speed of 1 µl per second during the exposure, minimizing radiation damage, and is suitable for sample volumes between 30 and 100 µl with larger volumes allowing for longer exposure times. In static mode samples are loaded and exposed without moving, and this enables data collections from samples with volumes as low as 15 µl. In this mode radiation damage is evident in protein samples in phosphate-buffered saline after 1 s, although this buffer system has been found to be particularly prone to radiation damage. The SEU can hold samples at temperatures ranging from 2 to 60°C for temperature-based exposures. In addition, the SEU maintains the sample capillary in a vacuum environment that removes the background due to atmospheric scatter. Static mode enables high-throughput data collection and well defined sample compositions for titrations of buffer components such as ligand concentrations *etc.* (McMahon *et al.*, 2020 ▸) or for time-dependent measurements (Kelly *et al.*, 2020 ▸). Typically, measuring a sample will take around 2 min in static mode, which includes 20–30 s for measurement and around 90 s for loading the sample and washing, rinsing and drying the sample cell. A 96-well plate full of samples can thus be measured in around 3 h.

#### 2.3.2. In-line size-exclusion chromatography coupled SAXS (SEC-SAXS)   

SEC-SAXS is a powerful technique for biological SAXS (bioSAXS) data collection, as it allows purification of the sample during the SAXS measurement (David & Pérez, 2009 ▸; Mathew *et al.*, 2004 ▸). The inherent difficulties associated with bioSAXS, namely buffer matching and polydispersity, are mitigated by SEC-SAXS as it allows for the effective separation of monomeric, oligomeric and aggregated structures (Durall *et al.*, 2020 ▸). SAXS measurements made across individual chromatographic peaks are averaged, producing a reliable measurement of a well defined particle [Fig. 5 ▸(*c*)]. This enables more detailed information to be obtained from complexes and the measurement of more unstable proteins, as well as ensuring complete buffer matching, thus improving the overall quality of the data. Sample volumes are typically in the range 25–50 µl at a flow rate of 160 µl per minute. Concentrations in the range 1–10 mg ml^−1^ are recommended for protein samples in SEC-SAXS mode, albeit dependent on the molecular weight of the particles under study. To minimize the impact of radiation damage, users are strongly advised to add 1–2% glycerol or sucrose to running buffers and to use organic buffering systems such as HEPES or Tris rather than phosphate.

SEC-SAXS on B21 uses a dual Agilent 1260 HPLC system [Fig. 6 ▸(*a*), (1)], with many different columns available on the beamline (Table 2 ▸) [Fig. 6 ▸(*a*), (3)]. The dual system allows for equilibration of one column while another is delivering sample to the beamline. With four-way buffer switching per pump and a common autosampler [Fig. 6 ▸(*a*), (2)], up to 96 samples can be queued to run on two columns with up to eight buffers. The inline UV–VIS detector [Fig. 6 ▸(*a*), (2)] can record up to eight wavelengths at a time in a wavelength range of 190–950 nm.

The system is partially integrated into the beamline control and analysis software. The injection and UV–VIS signals are monitored, and a failure of the beamline such as a storage-ring dump will cause the HPLC system to stop. However, there is no single interface controlling the beamline and the HPLC. The sample cell can also be changed to a specialized synthetic mica cell with an ∼500× reduction in background scattering. Switching between the HPLC system and the BIOSAXS robot is rapid (<5 min).

An offline multi-angle light scattering (MALS) instrument is also available on the beamline. MALS provides an independent scattering-based measurement of the size, polydispersity and molecular weight of samples along with SAXS data (Behrens *et al.*, 2020 ▸; Song *et al.*, 2020 ▸).

#### 2.3.3. Manual loading   

On beamline B21, it is possible to measure viscous solutions, solids and hydrogels into a custom multi-purpose sample (MPS) cell for measurement [Fig. 6 ▸(*b*)] (Edwards-Gayle *et al.*, 2020 ▸). Samples can be loaded into 2 mm path-length polyimide capillaries or into wells on 3D printed sticks (both available from the beamline). The temperature of the MPS cell can be controlled between 4 and 60°C. The MPS cells have been used to measure a variety of samples such as gels (McAulay *et al.*, 2020 ▸), lipids (Yeh *et al.*, 2020 ▸) and peptide self-assembly (Pelin *et al.*, 2020 ▸).

Finally, a peristaltic pump and two syringe pumps running under the beamline control system provide additional opportunities for loading and recirculating samples with stepwise addition of reaction components for following enzymatic or chemical processes *in situ*.

#### 2.3.4. End-station vacuum system   

All SAXS measurements are made with the sample cell maintained in a high vacuum, where the SAXS camera is formed from a contiguous vacuum system starting from the silicon nitride window (30 m from the source) to the detector (40 m from the source) (Fig. 3 ▸). This constitutes a large evacuated volume (∼750 l) that would prohibit rapid changing of sample cells. Therefore, to minimize routine evacuation of the entire camera section we have installed a set of vacuum gate valves to isolate the BIOSAXS SEU sample section (Fig. 4 ▸). These valves are interlocked with the experimental hutch door and vacuum gauges. The valves close automatically when the beamline door is open or if the camera pressure exceeds a threshold. Likewise, the valves will not open if the isolated sample-section vacuum cannot reach a predetermined value. The isolated sample section can be vented and pumped down in minutes to facilitate cell changes.

## 3. Primary data reduction   

Beamline control is provided by the *Generic Data Acquisition*, *GDA*, software developed at Diamond Light Source (http://www.opengda.org/). *GDA* is used to control experiments and collect data that are stored in the NXS file format (Könnecke *et al.*, 2015 ▸). An automated data normalization and reduction pipeline is triggered for each NXS file using routines in the Diamond Light Source developed *Data Analysis WorkbeNch* (*DAWN*) (Basham *et al.*, 2015 ▸). The output is azimuthally averaged normalized one-dimensional data in a three column ASCII file (*.dat). For the SHR data, further processing is triggered by custom Python scripts developed on B21 that automatically average, subtract and eliminate dead frames due to air or radiation damage.

For the HPLC data, images are reduced to *.dat files but assessments of polydispersity across elution peaks and selection of background and sample frames for averaging and subtraction are done manually. The beamline primarily uses the JAVA-based program *ScÅtter* (http://www.bioisis.net) for data analysis, for which there are tutorials online. *ScÅtter* allows users to process integrated and normalized data, perform background subtractions, calculate primary SAXS invariants such as radius of gyration, volume of correlation, Porod–Debye exponent and particle volume, and perform indirect Fourier transform.

## 4. Facility access   

Access to beamline B21 occurs through either PRP (peer-review panel) or rapid access proposals. PRP access can be combined with MX (macromolecular crystallography) for combined access to multiple beamlines through a Block Allocation Group (BAG) application (https://uas.diamond.ac.uk). Calls for these proposals occur biannually. Rapid access proposals can be submitted at any time during the year and will be reviewed by the beamline staff. In addition, Diamond offers proprietary access to industrial clients.

## 5. Mail-in   

Many solution-state measurements can be performed without users directly on the beamline. Here, B21 has pioneered a mail-in service for United Kingdom and European Union users. Users that are registered with Diamond Light Source and are MX or soft-condensed matter (SM) users will receive periodic emails notifying them of potential mail-in time. Time is awarded on a first-come first-served basis. There are usually 15 user slots per mail in session, with a maximum of 16 samples per user. It is also possible to run BAG, PRP or rapid access proposals as mail-in sessions upon request.

## 6. Science highlights   

### 6.1. Zinc finger protein CNBP alters the 3D structure of lncRNA Braveheart in solution (Kim *et al.*, 2020 ▸)   

Long non-coding RNAs (LnRNAs) are of interest due to their importance in many biological processes, including histone modification, chromatin dynamics, gene expression and genetic imprinting. LnRNAs perform these functions through interaction with other molecules (including proteins, DNA and other RNA molecules). A challenge in this field is to understand LnRNA–ligand interaction through biophysical techniques. Many techniques for probing 3D structures, for example NMR and crystallization, have proved challenging due to the size and flexibility of LnRNA molecules. Here, Kim *et al.* (2020 ▸) focused on the structural determination of Braveheart (Bvht) RNA, which binds cellular nucleic acid binding protein (CNBP) and then binds to a component of the Polycomb repressive complex (PRC2) which alters chromatin modification.

**Figure 7:**
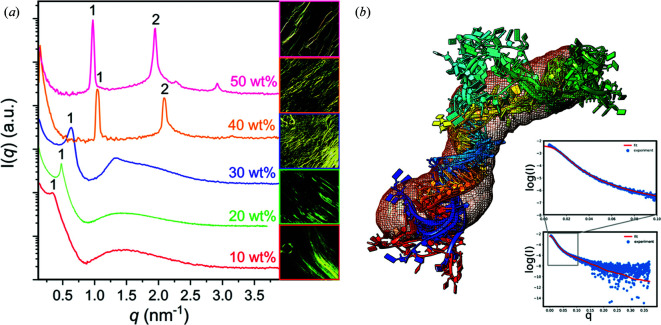
(*a*) SAXS data collected using the MPS cell, and polarized optical light micrographs of lyotropic liquid crystal phases of an azo­benzene photosurfactant at a variety of concentrations. [From Houston *et al.* (2019 ▸). Reproduced by permission of The Royal Society of Chemistry (https://www.rsc.org/)]. (*b*) An atomistic model of a portion of a long non-coding RNA (Braveheart) superimposed on a low-resolution envelope generated *de novo* from SAXS data. The fit of the atomistic model to the SAXS data is shown in the inset. [Reproduced from Kim *et al.* (2020 ▸)].

Inline SEC-SAXS data were collected on B21 from both full-length Bvht RNA and fragments of Bvht RNA. It has a defined 3D structure that is flexible, and forms complexes with protein CNBP through interactions at multiple domains, leading to compaction in the RNA structure. SAXS data for Bvht were used to assess models produced by the *ERWIN RNA* modelling software, for which there was good agreement [Fig. 7 ▸(*b*)]. This paper highlights the advantage of using SAXS combined with computer modelling applications in the field of RNA and RNA systems that can be challenging to study using techniques such as crystallography.

#### 6.1.1. Multimodal control of liquid crystalline mesophases from surfactants with photoswitchable tails (Houston *et al.*, 2019 ▸)   

Lytrophic liquid crystalline phases (LLC), derived from concentration-dependant self-assembly of surfactants, are being developed for a variety of applications including drug-delivery vessels, optical modulators or templates for the construction of porous materials. There is particular interest in the design of functionalized LLCs, for example photo-responsiveness. Azobenzene undergoes *trans*–*cis* photo-isomerization under UV irradiation, allowing it to act as a prototypical molecular switch.

Houston *et al.* (2019 ▸) used the B21 MPS cell to obtain SAXS data that showed the ability of azobenzene photosurfactants (AzoPS) to form LLC phases. The SAXS data allowed direct assignment of the phase symmetry of the surfactants, with the most common being hexagonal-phase and lamellar-phase symmetry, depending upon the alkyl tail groups and polar head groups of the AzoPS’s, in both the *cis* and *trans* forms [Fig. 7 ▸(*a*)]. This work eloquently demonstrates SAXS as a powerful technique to assign phases in order to further the understanding of how to fine tune the assembly of LLC phases.

## 7. Conclusions and future directions   

SAXS experiments on biological particles in solution (bioSAXS) are often made under dilute conditions and measure very small differences. Since the bioSAXS community represents the largest user community for B21, we have optimized the beamline to provide the lowest possible instrumentation background. As the X-ray beam enters the beamline, it will encounter three reflective surfaces due to the monochromator and mirror, four sets of slits, a silicon nitride window that separates ultra-high from low-vacuum regions, and the beamstop. Significant reductions in background were realized by optimizing the distances between the SiN window and the detector, and the beamstop and the detector. Focusing using the toroidal mirror is achieved by adjusting the bend of the mirror through the midpoint. A systematic scan of the X-ray beam across the surface demonstrated non-ideal curvature near the edges of the mirror. This contributes to instrumentation background if the mirror is fully illuminated. Therefore, we optimized further by only illuminating the properly bent regions of the mirror by adjusting the upstream slits. An upgrade of the mirror support and bending mechanism is underway to eliminate these distortions.

An in-vacuum sample cell, well separated germanium-edged slits, an in-vacuum photon-counting detector and a single window made of micrometre-thick silicon nitride placed as far from the sample as possible combine to make B21 a low-background SAXS beamline suitable for the measurement of weakly scattering samples. Low setup overheads and a high level of sample delivery automation make it particularly useful for bioSAXS experiments. The most recent developments on B21 have been the initiation of a mail-in service and the measurement of solid and viscous samples via a custom MPS cell. Developments in the near future will focus on these capabilities.

Running samples via mail-in requires investment in logistics. We are working to create an interface such that users can register their samples via *iSPyB/Synchweb* (De Maria Antolinos *et al.*, 2015 ▸; Fisher *et al.*, 2015 ▸) such that the identity, location, data and metadata of samples can be tracked throughout the process of shipment, storage, collection, data processing and retrieval of data by the user. In addition, the inline HPLC system will be upgraded such that it runs fully under the control of the beamline control software. This will allow data collection to be automatically stopped and started in response to factors such as loss of beam in the storage ring and for interleaving of samples being run via the BIOSAXS robot and via the HPLC system.

The measurement of solid and viscous samples is currently somewhat laborious, as capillaries and sample sticks must be applied to the MPS cell by hand, which involves a user entering and then searching and securing the experimental hutch. There is a need for automated loading of such samples, for example by a robotic arm. Finally, addition of a WAXS detector will improve our ability to measure structure factor peaks from samples such as polymers.

A future upgrade of B21 will introduce pink-beam capabilities to the beamline where the first crystal of the DMM will provide a large energy bandpass X-ray beam to the sample position for future time-resolved SAXS (Kirby & Cowieson, 2014 ▸), X-ray foot-printing (Gupta *et al.*, 2016 ▸) and diffracted X-ray tracking experiments (Shinohara *et al.*, 2013 ▸).

## Figures and Tables

**Table 1:** Beamline details

Beamline name	B21, biological X-ray scattering
Source type	Bending magnet
Monochromator	Double multi-layer monochromator
Energy range	9.5–14 keV
Wavelength range	0.89–1.3 Å
Mirrors	1.2 m platinum-coated Si toroidal mirror
Beam size at focal point at detector (FWHM)	34 × 40 µm (horizontal × vertical)
Beam size at sample (FWHM)	1102 × 240 µm
Camera length	3.7 m
Detector	Eiger 4M (Dectris)
*Q* range	0.0026–0.34 Å^−1^

**Table 2:** Size-exclusion columns available on B21

Column	Volume (ml)	Mass range (kDa)	Maximum pressure (bar†)	Recommended flow rate (ml min^−1^)
Superdex 75 (GE Healthcare)	2.4	3–70	35	0.075
Superdex 200 (GE Healthcare)	2.4	100–600	35	0.075
Superose 6 (GE Healthcare)	2.4	5–5000	35	0.075
KW-402.5 (Shodex)	4.6	5–100	85	0.16
KW-403 (Shodex)	4.6	10–700	85	0.16
KW-404 (Shodex)	4.6	30–4000	85	0.16
KW-405 (Shodex)	4.6	40–2000	85	0.16

† 1 bar = 100 000 Pa.
